# Prehabilitation in patients awaiting elective coronary artery bypass graft surgery – effects on functional capacity and quality of life: a randomized controlled trial

**DOI:** 10.1177/0269215520933950

**Published:** 2020-06-16

**Authors:** Carolin Steinmetz, Birna Bjarnason-Wehrens, Heike Baumgarten, Thomas Walther, Thomas Mengden, Claudia Walther

**Affiliations:** 1 Institute of Sport Science, Department of Training Science and Kinesiology, University of Göttingen, Göttingen, Germany; 2 Institute of Cardiology and Sports Medicine, German Sport University Cologne, Cologne, Germany; 3 Kerckhoff Heart Center, Department of Cardiothoracic Surgery, Bad Nauheim, Germany; 4 Department of Cardiothoracic Surgery, University of Frankfurt, Frankfurt am Main, Germany; 5 Kerckhoff Heart Center, Department of Rehabilitation, Bad Nauheim, Germany; 6 Department of Cardiology, University of Frankfurt, Frankfurt am Main, Germany

**Keywords:** Coronary artery disease, cardiac rehabilitation, coronary artery bypass surgery, quality of life, aerobic exercise

## Abstract

**Objective:**

To determine the impact of an exercise-based prehabilitation (EBPrehab) program on pre- and postoperative exercise capacity, functional capacity (FC) and quality of life (QoL) in patients awaiting elective coronary artery bypass graft surgery (CABG).

**Design:**

A two-group randomized controlled trail.

**Setting:**

Ambulatory prehabilitation.

**Subjects:**

Overall 230 preoperative elective CABG-surgery patients were randomly assigned to an intervention (IG, *n* = 88; *n* = 27 withdrew after randomization) or control group (CG, *n* = 115).

**Intervention:**

IG: two-week EBPrehab including supervised aerobic exercise. CG: usual care.

**Main measures:**

At baseline (T1), one day before surgery (T2), at the beginning (T3) and at the end of cardiac rehabilitation (T4) the following measurements were performed: cardiopulmonary exercise test, six-minute walk test (6MWT), Timed-Up-and-Go Test (TUG) and QoL (MacNew questionnaire).

**Results:**

A total of 171 patients (IG, *n* = 81; CG, *n* = 90) completed the study. During EBPrehab no complications occurred. Preoperatively FC (6MWT_IG_: 443.0 ± 80.1 m to 493.5 ± 75.5 m, *P* = 0.003; TUG_IG_: 6.9 ± 2.0 s to 6.1 ± 1.8 s, *P* = 0.018) and QoL (IG: 5.1 ± 0.9 to 5.4 ± 0.9, *P* < 0.001) improved signiﬁcantly more in IG compared to CG. Similar effects were observed postoperatively in FC (6MWD_IG_: Δ-64.7 m, p_T1–T3 = _0.013; Δ+47.2 m, p_T1–T4 < _0.001; TUG_IG_: Δ+1.4 s, p_T1–T3 = _0.003).

**Conclusions:**

A short-term EBPrehab is effective to improve perioperative FC and preoperative QoL in patients with stable coronary artery disease awaiting CABG-surgery.

ID: NCT04111744 (www.ClinicalTrials.gov; *Preoperative Exercise Training for Patients Undergoing Coronary Artery Bypass Graft Surgery- A Prospective Randomized Trial*)

## Introduction

Within the last decade, the number of older patients undergoing heart surgery has increased dramatically. In 2016, 60% of all (*n* = 72.761) coronary artery bypass graft (CABG) surgeries performed in Germany involved patients aged 65–80 years.^1^

In this population, the prevalence of comorbidities and frailty is high. Previous studies demonstrated, a poor physical health status prior to CABG-surgery to be associated with longer hospital stay, prolonged postoperative ventilation and higher incidence of perioperative morbidity and mortality.^2–4^ Especially in old and frail patients, reduced physical fitness and the loss of physical functioning prior, during and after the hospital stay have a large negative effect.^5^

Therefore, interventions to counteract those preconditions such as prehabilitation have increasingly attracted attention. Prehabilitation programs aim to increase the patients physical fitness and functional capacity prior to surgery in order to improve his/her postoperative recovery as well as the efficacy of the following cardiac rehabilitation.^6–8^ Furthermore, such programs may be able to reduce the long-term need for care and improve the patients ability of independent living.^9,10^

Until now only few studies, including low risk patients, have evaluated the safety and efficacy of cardiac prehabilitation.^11–15^ The aim of this prospective randomized controlled study was to evaluate the effects of a two-week exercise-based prehabilitation program on the pre- and postoperative outcomes on cardiopulmonary fitness, fuctional capacity and quality of life in patients with stable coronary artery disease awaiting elective CABG-surgery.

## Methods

The “Preoperative Excercise Training for Patients Undergoing Coronary Artery Bypass Graft Surgery- A Prospective Randomized Trial” (ID: NCT04111744, www.ClinicalTrials.gov) was designed as a single center non-blinded prospective randomized controlled study with parallel assignment. The study protocol was approved by the responsible ethic committee in Gießen, Germany (Code: DE/HKHE20). All patients gave written informed consent. This study was conducted according to the principles of good clinical practice. German Heart Research Foundation, Wiliam G. Kerckhoff Foundation and Willy Robert Pitzer Foundation supported this study. Kerckhoff Heart Center, Bad Nauheim, Germany conducted the trial in cooperation with the German Sport University Cologne, Germany and the Justus-Liebig-University Gießen, Germany. The results presented here are partial outcomes of the main study “The Preoperative Excercise Training for Patients Undergoing Coronary Artery Bypass Graft Surgery- A Prospective Randomized Trial”. Other outcomes like effects of short-term exercise-based prehabilitation on hemodynamics as well as clinical and surgical parameters will be published soon.

During the period from December 2014 to March 2018, 824 eligible patients were screened for participation into the study in Kerckhoff Heart Center, Bad Nauheim, Germany.

Inclusion critera were: indication for CABG-surgery according to the guidelines of the European Heart Journal,^16^ the German Cardiac Society^17^ and the American College of Cardiology Foundation/American Heart Association,^18^ stable coronary artery disease, exercise-induced angina pectoris threshold ⩾50 W and written informed consent for participation in the study.

Exclusion critera were: unstable angina pectoris and/or myocardial infarction during the last two weeks, left main stem stenosis ⩾50%, exercise-induced angina threshold or ischemia at <50 W, left ventricular ejection fraction <35%, significant ventricular arrhythmia or relevant heart valve disease, myocarditis, hypertrophic obstructive cardiomyopathy, CABG-surgery during the last six months, peripheral arterial disease Fontaine ⩾IIb, orthopedic or neurologic preconditions precluding exercise training as well as the unability to attend the prehabilitation program due to physical limitations or long distance to cardiac rehabilitation (one way: >60 minutes by driving).

**Figure 1.**
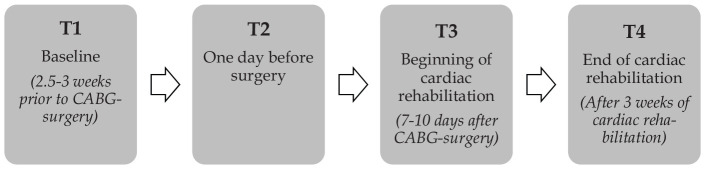
The defined study timepoints. CABG: Coronary artery bypass graft.

Study measurements were performed at baseline, one day before surgery, at the beginning and at the end of cardiac rehabilitation (Figure 1). All measurements were conducted by certified staff members (cardiologists, heart surgeons, exercise physiologists or nursing staff). At baseline medical history and cardiovascular risk factors as well as medication were recorded. At each study timepoint anthropometric data and blood pressure were assessed, blood samples were taken for further analysis and echocardiography was performed. In addition, patients completed a cardiopulmonary bicycle exercise test (spiroergometry), a six-minute walk test, a Timed-Up-and-Go Test and filled out the MacNew questionnaire.

A submaximal cardiopulmonary exercise test was performed because of the patients’ diagnosis (symptomatic coronary artery disease). The test was conducted on a bicycle ergometer using a ramp protocol starting with 50 W, and gradual increase by 10 W per minute until patients were subjectively exhausted or defined criteria for termination occurred.^19^ Peak workload (Watt and Watt/kg) and peak oxygen consumption (VO_2_peak L/min and mL/kg per minute) were assessed.

The six-minute walk test was conducted according to the American Thoracic Society guidelines.^20^ Improvement in six-minute walking distance ⩾50 m was defined as the minimal clinically important difference.^21^ The predicted values for the six-minute walk distance for the study population was 637.5 m.^22^ During the Timed-Up-and-Go Test patients were asked to rise from a 43-cm-high chair, walk as fast as possible to an identification mark on the floor 3 m away from chair, turn, walk back, and sit down again.^23^ The time needed to carry out the task was documented.

The MacNew questionnaire was used to assess quality of life. It is a specifically designed questionnaire for individuals with heart disease. It includes 27 items with different subscales (emotional, physical, social and global).^24^ Improvements ⩾0.5 points exceed the minimal clinically important difference.^25^

Patients randomized to the intervention group participated in a two-week preoperative exercise program including an individualized supervised and monitored cycle ergometer training three times per week. Individual exercise intensity was 70% of peak oxygen uptake (VO_2_peak). Every training session included two aerobic exercise workouts with a 15-minute phase of light gymnastics in between. The aerobic exercises started with two 10 minutes cycling workouts (1st session) which were gradually increase up to two 25 minutes cycling (6th session) in the course of the program (2nd and 3rd session: 2 × 15 minutes, 4th and 5th session: 2 × 20 minutes). The light gymnastic program included breathing techniques and coordination exercises on a chair. Adverse events like exercise-induced arrhythmias, unstable angina, fatal or non-fatal myocardial infarction and hospitalization were documented during the exercise-based prehabilitation program by the supervising medical staff. The control group received no preoperative training or further information. The usual care was provided by the patients’ general practitioner. Postoperatively all patients of both groups participated in a three-week cardiac rehabilitation program.

As the primary endpoint of this study was the change of endothelial function, the sample size for “The Preoperative Excercise Training for Patients Undergoing Coronary Artery Bypass Graft Surgery- A Prospective Randomized Trial” was calculated using the expected change in the results of the mean EndoPAT^®^-index by G-Power software version 3.1 (University of Düsseldorf, Germany). The calculation included a one-sided *t*-test to detect mean differences between two independent groups based on: 0.5 standard deviation, 0.5 effect size, 5% α-error and 95% power. The system computed a sample size of 88 participants in each group. In addition to our calculation the results of the pilot study from Ozasa et al.^26^ were taken into account. By using the standard deviation in the EndoPAT^®^-index of 0.56 for a study with two independent groups and a power of 95% the authors calculated a sample size of 226 participants to detect clinically meaningful changes in EndoPAT^®^-index after conventional aerobic endurance training in older heart failure patients (mean age: 79.5 years). Based on this and accounting for an expected loss to follow-up of 20%, 230 patients needed to be randomized in our trial.

After baseline assessment patients were randomly assigned to either prehabilitation or standard therapy before CABG by drawing an envelope with the treatment assignment enclosed from a closed box with mixed envelopes. Patient enrolment, randomization, and assignment to the intervention group was performed by two clinical investigators (CW and CS). Medical treatment was adjusted according to current clinical guidelines and was continued by the patients’ private physicians. The statistical analysis was performed as per protocol analysis consisting of all patients who completed all measurements at baseline. Continuous and categorial variables are presented by mean ± standard deviation with absolute and relative frequencies, respectively. Two-group comparisons of baseline variables were performed using Student’s *t*-test and Chi-square-test of independence for continuous and categorical variables. Multivariant analysis of variance with repeated measurements was used for statistical analyses of time-, group-, and treatment-related changes and differences, with *P* < 0.05 considered to be significant. In addition, the post-hoc analysis with Bonferroni correction was used. Statistics were calculated using SPSS^®^ version 21 (SPSS Inc., Cary, NC, USA).

## Results

**Figure 2.**
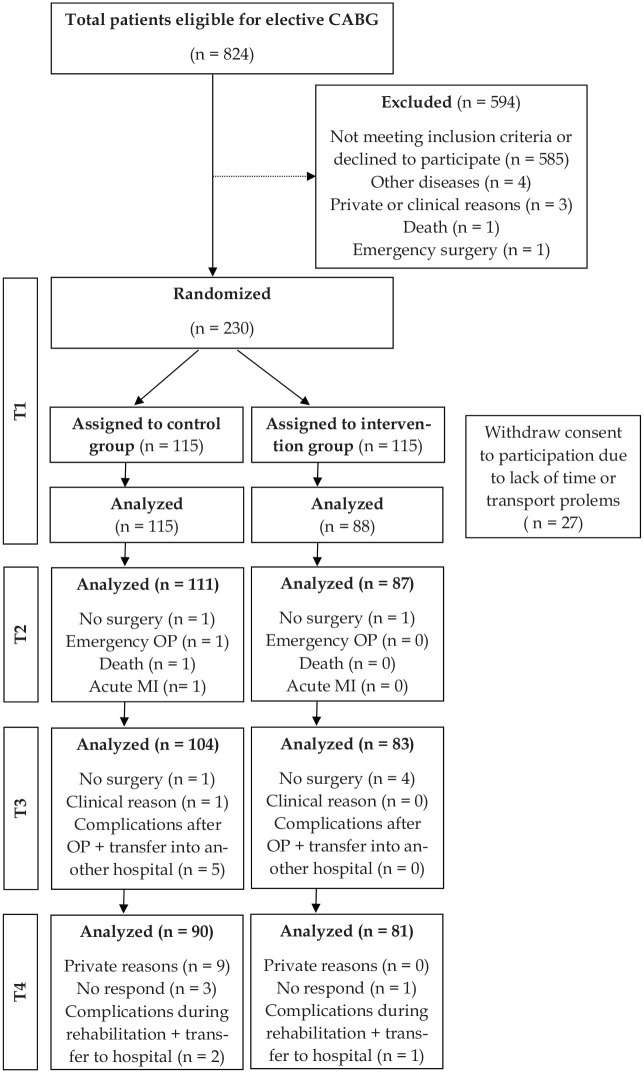
Study flow chart. CABG: Coronary artery bypass graft; T1: Baseline; T2: One day before surgery; T3: Beginning of cardiac rehabilitation; T4: End of cardiac rehabilitation; OP: Operation; MI: Myocardinfarction.

A total of 230 patients were randomized into the intervention group (*n* = 115) or control group (*n* = 115). After randomization 27 patient of the intervention group withdraw their consent to participate in the prehabilitation program due to lack of time or transport problems. Finally, 182 men and 21 women (67.1 ± 8.4 years; intervention group: *n* = 88; control group: *n* = 115) were included into the study (Figure 2). The baseline characteristics of the patient cohort are summarized in Table 1. There were no significant differences between the groups. Of the 203 participants enrolled in the study at baseline, 171 completed the study (intervention group: *n* = 81, control group: *n* = 90) (Figure 2).

**Table 1.** Baseline characteristics of the study population.

Characteristic	All (*n* = 203)	Intervention group (*n* = 88)	Control group (*n* = 115)	*P*-value
	*(mean ± SD)*	*(mean ± SD)*	*(mean ± SD)*	
Age (years)	67.1 ± 8.4	66.1 ± 9.0	67.9 ± 7.9	0.127^*^
Height (cm)	1.75 ± 8.1	1.74 ± 8.6	1.75 ± 7.7	0.294^*^
Weight (kg)	87.8 ± 15.2	88.2 ± 16.1	87.5 ± 14.4	0.758^*^
Body mass index (kg/m^2^)	28.7 ± 4.4	29.1 ± 4.7	28.5 ± 4.2	0.372^*^
Coronary artery disease (n)				
One-vessel coronary artery disease	10 (4.9%)	3 (3.4%)	7 (6.1%)	0.382^#^
Two-vessel coronary artery disease	37 (18.2%)	15 (17%)	22 (19.1%)	0.703^#^
Three-vessel coronary artery disease	156 (76.8%)	70 (79.5%)	86 (74.8%)	0.425^#^
LV ejection fraction (%)	56.1 ± 7.6	55.8 ± 7.2	56.2 ± 8.0	0.342^*^
CCS-classification (n)				
no angina pectoris	90 (44.3%)	37 (42%)	53 (46.1%)	0.566^#^
CCS I	11 (5.4%)	4 (4.5%)	7 (6.1%)	0.631^#^
CCS II	78 (38.4%)	38 (43.2%)	40 (34.8%)	0.223^#^
CCS III	15 (7.4%)	6 (6.8%)	9 (7.8%)	0.786^#^
Cardiovascular risk factors (n)				
Hyperlipidemia	175 (86.2%)	77 (87.5%)	98 (85.2%)	0.640^#^
Hypertension	178 (87.7%)	81 (92%)	97 (84.3%)	0.098^#^
Diabetes mellitus	74 (36.5%)	29 (33%)	45 (39.1%)	0.600^#^
Familial disposition	144 (70.9%)	65 (73.9%)	79 (68.7%)	0.422^#^
Smoking habits				
Never smoked	77 (37.9%)	34 (38.6%)	43 (37.4%)	0.856^#^
Ex-smoker	116 (57.1%)	49 (55.7%)	67 (58.3%)	0.713^#^
Smoker	10 (4.9%)	5 (5.7%)	5 (4.3%)	0.663^#^
Medical history				
Previous myocardial infarction (n)	65 (32%)	34 (38.6%)	31 (27%)	0.077^#^
Heart failure (n)				
NYHA I	4 (2%)	2 (2.3%)	2 (1.7%)	0.786^#^
NYHA II	41 (20.2%)	17 (19.3%)	24 (20.9%)	0.785^#^
NYHA III	10 (4.9%)	5 (5.7%)	5 (4.3%)	0.663^#^

cm: centimeter; kg: kilogram; m^2^: square meter; n: number; LV: left ventricular; CCS: Canadian Cardiovascular Society; NYHA: New York Heart Association; SD: standard deviation; ^*^: Independent *t*-test; ^#^: Chi-square-test.

There were no statistical significant changes in results of cardiopulmonary exercise test in the pre- and postoperative period (Table 2).

**Table 2.** Effects of two-week exercise-based prehabilitation prior to coronary artery bypass graft surgery on pre- and postoperative functional capacity, exercise capacity and quality of life.

	Intervention group				Control group				*P*-value			post-hoc^1^ (time)					
	T1	T2	T3	T4	T1	T2	T3	T4	Time	Group	Interaction	T1 vs T2	T2 vs T3	T3 vs T4	T1 vs T3	T1 vs T4	T2 vs T4
	*(mean ± SD)*				*(mean ± SD)*												
**Functional capacity**																	
6MWD (m)	443.0 ± 80.1	493.5 ± 75.5	378.3 ± 109.5	490.2 ± 85.2	445.6 ± 105.6	459.8 ± 110.1	344.8 ± 105.6	451.3 ± 106.4	*P* < 0.001	*P* = 0.063	*P* = 0.003	*P* < 0.001	*P* < 0.001	*P* < 0.001	*P* < 0.001	*P* < 0.001	*P* = 1.000
TUG (s)	6.9 ± 2.0	6.1 ± 1.8	8.3 ± 2.6	6.4 ± 2.1	7.4 ± 2.4	7.3 ± 2.5	10.0 ± 4.0	7.5 ± 2.4	*P* < 0.001	*P* = 0.001	*P* = 0.018	*P* < 0.001	*P* < 0.001	*P* < 0.001	*P* < 0.001	*P* = 0.853	*P* = 0.400
**Maximum workload**																	
Watt	97.7 ± 23.2	100.6 ± 22.3	77.8 ± 22.6	96.0 ± 28.3	100.4 ± 22.6	99.3 ± 23.8	74.3 ± 19.0	92.9 ± 21.1	*P* < 0.001	*P* = 0.723	*P* = 0.201	*P* = 1.000	*P* < 0.001	*P* < 0.001	*P* < 0.001	*P* = 0.056	*P* = 0.008
Watt/kg	1.15 ± 0.31	1.18 ± 0.30	0.93 ± 0.28	1.16 ± 0.37	1.19 ± 0.31	1.18 ± 0.32	0.89 ± 0.25	1.12 ± 0.28	*P* < 0.001	*P* = 0.827	*P* = 0.160	*P* = 1.000	*P* < 0.001	*P* < 0.001	*P* < 0.001	*P* = 1.000	*P* = 0.326
**Peak oxygen consumption**																	
L/min	1326.7 ± 328.4	1373.1 ± 346.0	1053.2 ± 359.4	1282.9 ± 381.5	1362.3 ± 311.5	1369.6 ± 326.1	1021.1 ± 263.7	1255.5 ± 315.7	*P* < 0.001	*P* = 0.907	*P* = 0.515	*P* = 1.000	*P* < 0.001	*P* < 0.001	*P* < 0.001	*P* = 0.038	*P* < 0.001
mL/kg per minute	15.7 ± 4.1	16.3 ± 4.4	12.5 ± 4.1	15.6 ± 4.8	16.2 ± 4.1	16.3 ± 4.0	12.3 ± 3.2	15.2 ± 3.8	*P* < 0.001	*P* = 0.964	*P* = 0.520	*P* = 0.921	*P* < 0.001	*P* < 0.001	*P* < 0.001	*P* = 0.625	*P* = 0.027
**Quality of life (preoperative)**																	
global	5.1 ± 0.9	5.4 ± 0.9			5.3 ± 1.0	5.3 ± 1.0			*P* < 0.001	*P* = 0.935	*P* < 0.001						
physical	5.0 ± 1.0	5.4 ± 1.0			5.2 ± 1.1	5.3 ± 1.1			*P* < 0.001	*P* = 0.745	*P* < 0.001						
emotional	5.2 ± 1.0	5.5 ± 1.0			5.3 ± 1.1	5.3 ± 1.1			*P* < 0.001	*P* = 0.785	*P* < 0.001						
social	5.2 ± 1.0	5.5 ± 1.0			5.4 ± 1.1	5.4 ± 1.1			*P* < 0.001	*P* = 0.957	*P* < 0.001						
**Quality of life**																	
global	5.2 ± 0.9	5.5 ± 0.8	4.8 ± 1.1	5.4 ± 1.1	5.3 ± 1.0	5.3 ± 1.1	4.7 ± 1.2	5.4 ± 1.0	*P* < 0.001	*P* = 0.732	*P* = 0.137	*P* < 0.001	*P* < 0.001	*P* < 0.001	*P* < 0.001	*P* = 0.158	*P* = 1.000
physical	5.1 ± 1.0	5.5 ± 0.9	4.5 ± 1.2	5.3 ± 1.1	5.3 ± 1.1	5.3 ± 1.2	4.5 ± 1.3	5.2 ± 1.2	*P* < 0.001	*P* = 0.748	*P* = 0.150	*P* < 0.001	*P* < 0.001	*P* < 0.001	*P* < 0.001	*P* = 1.000	*P* = 1.000
emotional	5.3 ± 1.0	5.6 ± 0.9	5.1 ± 1.1	5.6 ± 1.2	5.4 ± 1.1	5.4 ± 1.1	5.0 ± 1.2	5.6 ± 1.1	*P* < 0.001	*P* = 0.608	*P* = 0.146	*P* = 0.001	*P* < 0.001	*P* < 0.001	*P* < 0.001	*P* = 0.066	*P* = 1.000
social	5.3 ± 0.9	5.6 ± 0.9	4.7 ± 1.2	5.5 ± 1.1	5.4 ± 1.1	5.4 ± 1.2	4.7 ± 1.3	5.4 ± 1.1	*P* < 0.001	*P* = 0.595	*P* = 0.141	*P* < 0.001	*P* < 0.001	*P* < 0.001	*P* < 0.001	*P* = 0.891	*P* = 1.000

1 Bonferroni correction (p < 0.05); vs: versus; 6MWD: 6-minute walk distance; TUG: Timed-Up-and-Go Test; T1: Baseline (2.5–3 weeks prior to CABG-surgery); T2: One day before surgery; T3: Beginning of cardiac rehabilitation (7–10 days after CABG-surgey); T4: End of cardiac rehabilitation (after 3 weeks of cardiac rehabilitation); SD: standard deviation; m: meter; s: seconds; L/min: liter per minute; mL/kg per minute: milliliter per kilogram per minute.

During the pre- and postoperative period, the six-minute walk distance and the Timed-Up-and-Go Time improved significantly in both groups. However, the changes in six-minute walk distance (intervention group: Δ+50.5 m, *P* < 0.001; control group: Δ+14.2 m, *P* < 0.001; *P* = 0.003) and Timed-Up-and-Go Time (intervention group: Δ–0.8 s, *P* < 0.001; control group: Δ–0.1 s, *P* < 0.001; *P* = 0.018) were preoperatively significant greater in the intervention group compared to control group. Similar effects were demonstrated in the postoperative period with more pronounced improvements in the intervention group compared to the control group (six-minute walk distance: “baseline” versus “beginning of cardiac rehabilitation”: intervention group: Δ–64.7 m; control group: Δ–100.8 m; *P* = 0.013; “baseline” versus “end of cardiac rehabilitation”: intervention group: Δ+47.2 m; control group: Δ+5.7 m; *P* < 0.001; Timed-Up-and-Go Test: “baseline” versus “beginning of cardiac rehabilitation”: intervention group: Δ+1.4 s; control group: Δ+2.6 s; *P* = 0.003) (Table 2).

As a result of the preoperative period, significant interaction between groups was seen in all domains of quality of life. In the intervention group, the improvements were more pronounced compared to control group (intervention group: Δ0.3–0.4, *P* ⩽ 0.001; control group: Δ0–0.1; *P* ⩽ 0.001; *P* < 0.001). However, in the postoperative period no significant intervention effect was observed (Table 2). The results vary because of a high postoperative dropout rate (Figure 2). No significant interaction was found in the main calculation over all study timepoints. Because of the significant post-hoc test during preoperative period additional calculations showed a preoperative significant interaction in all domains of quality of life (Table 2).

The exercise-based prehabilitation program was very well tolerated by all patients of intervention group. No exercise-related complications occurred.

## Discussion

The main results of this prospectively randomized trial demonstrate that a two-week exercise-based prehabilitation program prior to CABG-surgery in older patients (>65 years) is effective to improve preoperative functional capacity (six-minute walk distance, Timed-Up-and-Go Time) and quality of life. In addition, it positively influences the postoperative results of cardiac rehabilitation on six-minute walk distance and Timed-Up-and-Go Time, however the short-term exercise-based prehabilitation program was not effective to improve cardiopulmonary exercise capacity. This is one of the first prehabilitation studies including patients with a higher-risk profile (three-vessel coronary artery disease: >75%; CCS II: >38%; see Table 1) prior to elective CABG-surgery. The results confirm the feasibility, safety and the efficacy of an individualized exercise-based prehabilitation program in this population.

Results of several studies show that cardiac rehabilitation after CABG-surgery is effective to increase exercise capacity,^27–29^ improve quality of life^28,30^ and reduce mortality.^31,32^ Older patients with multimorbidity have the greatest benefit of cardiac rehabilitation.^28,33^ However, only few studies have evaluated the effect of prehabilitation prior to CABG-surgery. In the meta-analysis of Snowden et al.^34^ including 17 studies (*N* = 2689) it could be demonstrated that a preoperative exercise intervention reduced postoperative pulmonary complications significantly. Moreover, the results show a significant decrease in length of hospital stay in older patients after preoperative exercise intervention.^34^ However, the quality of this meta-analysis is influenced by the different study-characters and the great variation of the preoperative interventions provided, especially regarding exercise modalities, intensity and duration of the preoperative interventions. Most of the recently published prehabilitation studies are pilot studies with a small sample size, including a non-supervised exercise intervention and/or mixed patient population prior to valve and CABG-surgery.^11–14,35^ Promising results are to be expected from an ongoing multicentre randomized controlled trial focusing on the prehabilitation of older patients (⩾65 years) undergoing heart surgery.^11^

It is well known, that postoperative exercise-based cardiac rehabilitation significantly improves six-minute walk distance in older patients.^28,36,37^ Our results demonstrate that a two-week exercise-based prehabilitation program significantly improve preoperative six-minute walk distance (intervention group: +50.5 m versus control group: +14.2 m; *P* = 0.003). The minimal clinically important difference^21^ of 50 m was only achieved in patients of the intervention group. These findings go well in line with the results of two earlier prehabilitation pilot studies on preoperative six-minute walk distance.^12,35^ Both studies demonstrated a significant improvement in six-minute walk distance. In the study of Sawatzky et al.^12^ (*N* = 17; randomized controlled trail, control group no exercise training) the mean improvement in six-minute walk distance was Δ+132 m (prehab baseline: 342 ± 79 m, prehab preoperative: 474 ± 101 m, *P* < 0.05), after a minimum of four weeks of (8.2 ± 2.2 weeks; 19 ± 7 exercise sessions) prehabilitation. The positive results of the prehabilitation could be still observed after three months’ post-surgery. The better results in the six-minute walk distance compared to our results may be explained by the significantly longer exercise duration and the mean age of the patients. In the study of Waite et al.^35^ (*N* = 22; cohort study; home-based intervention ⩾6 weeks) the improvements on six-minute walk distance were less pronounced (+42.5 m ± 27.8 m), probably due to the older age of the participants (>65 years) and/or the home-based setting.

At admittance to cardiac rehabilitation the six-minute walk distance in our study population was longer compared to other cardiac rehabilitation studies post cardiac surgery.^28,36,37^ This may on one hand be due to the technical progress and improvement in cardiac surgery, but on the other hand it could be a result of the prehabilitation program. Furthermore, our study demonstrates more pronounced improvements in six-minute walk distance in the intervention group compared to control group (“baseline” versus “beginning of cardiac rehabilitation”: *P* = 0.013; “baseline” versus “end of rehabilitation”: *P* < 0.001) in the postoperative period. Finally, the predicted six-minute walk distance for the study population of 637.5 m was not achieved in both groups during all study timepoints.

As a result of the exercise-based prehabilitation program the reduction in preoperative Timed-Up-and-Go Time was significantly more pronounced in the intervention group compared to the control group (–0.8 s vs –0.1 s; *P* = 0.018). These preoperative improvements were, at least partly, maintained in the postoperative period (“baseline” vs “beginning of cardiac rehabilitation”: intervention group: Δ+1.4 s; control group: Δ+2.6 s; *P* = 0.003). No other currently published cardiac prehabilitation study used the Timed-Up-and-Go Test to assess the efficacy of a preopartive intervention in improving mobility.

The findings of the present study confirm the positive effect of prehabilitation on six-minute walk distance and Timed-Up-and-Go Time and underline the efficacy of exercise-based cardiac rehabilitation in improving six-minute walk distance and Timed-Up-and-Go Time post CABG-surgery.

The results of the present study demonstrate significant improvements in all domains of quality of life after a two-week prehabilitation, being more pronounced in intervention group compared to control group (intervention group: Δ0.3–0.4, *P* ⩽ 0.001; control group: Δ0–0.1; *P* ⩽ 0.001; *P* < 0.001). Nevertheless, the minimal clinically important difference of 0.5 points^25^ was not achieved. Pfaffenberger et al.^38^ published reference scores from patients 1–4 days prior CABG-surgery measured by MacNew questionnaire. In comparison to those scores, the results of our study population were up to 0.4 points higher, which demonstrates the positive effect of the prehabilitation program. However, no relevant effect was obvious in the postoperative period.

Similar positive effects of a prehabilitation program on quality of life could be observed by Arthur et al.^15^ with a significant improvement (mean change during waiting period: intervention group: 9.46 ± 34.39, control group: –2.06 ± 33.70; *P* = 0.01) of quality of life using the SF-36 questionnaire in intervention group compared to control group after an eight-week prehabilitation.

One of the main goals of cardiac rehabilitation is to increase exercise capacity.^39,40^ In the present study no intervention effects on exercise capacity could be demonstrated, neither pre- or postoperatively. These results are comparable to those of Arthur et al.,^15^ who also did not find an improvement of exercise capacity after an eight-week prehabilitation. Baseline exercise capacity was similar compared to our study. Reasons for missing effects of the prehabilitation program of our study on exercise capacity may be the short duration of the exercise program or the missing multimodal prehabilitation approach in our trial. Carli et al.^41^ did see positive effects during a multimodal and prolonged exercise-based prehabilitation on exercise capacity.

The present study has some limitations. Main limitation in this trial is the high dropout rate of 27 subjects of the intervention group. Reasons for withdrawal were lack of time or transport problems. The dropout rate during the study period was 25.7% and therefore higher than the 20% assumed by calculating the sample size. Therefore, the results and effects of the intervention program have to be interpreted with caution.

Another limitation is the lack of blinding of the study population and the study investigators. However, it is in the nature of the intervention in exercise trials that study participants cannot be blinded. According to the setting and design of this single center trial blinding of the study investigators could not be provided.

Furthermore, it cannot be excluded that patients in the control group were impacted by the “Hawthorn effect” and the results in the control group may have been influenced by this effect.^42^

In addition, the duration of the prehabilitation program may have been too short to induce changes in the primary endpoint peak oxygen uptake. But the relatively short waiting time in Germany did not allow to enlarge the prehabilitation period. Although the feasability of a prehabilitation with a multimodal combined program, including psychological treatment, individualized resistance and aerobic exercise training is necessary to determine.

In conclusion, a short-term endurance training in older patients (>65 years) with stable coronary artery disease awaiting CABG-surgery is feasible, safe and effective to improve preoperative functional capacity (six-minute walk distance, Timed-Up-and-Go Time), and quality of life. It was even effective to improve postoperative results in cardiac rehabilitation on functional capacity. The prehabilitation did not influence exercise capacity. In order to prevent or reduce the need for care and to re-establish the possibility of independent living, pre- and postoperative rehabilitation in older patients should focus on the enhancement in functional capacity.

**Clinical messages**

A short-term exercise-based prehabilitation in older patients with stable coronary artery disease awaiting CABG-surgery is effective to improve preoperative functional capacity and quality of life. The focus of exercise-based prehabilitation should be set on enhancement of functional capacity to improve postoperative recovery and to increase efficacy of rehabilitation programs.
